# A273 INCREASING THE REPRESENTATION OF INDIANS IN MICROBIOME RESEARCH: HOW DOES THEIR GUT MICROBIOME DIFFER?

**DOI:** 10.1093/jcag/gwac036.273

**Published:** 2023-03-07

**Authors:** L D D'Aloisio, C McComb, V Shetty, M Ballal, S Ghosh, D L Gibson

**Affiliations:** 1 Biology, University of British Columbia Okanagan Campus, Kelowna, Canada; 2 Microbiology, Kasturba Medical College Manipal, Enteric Diseases; 3 Microbiology, Kasturba Medical College, Manipal, India; 4 Biochemistry and Molecular Biology, University of British Columbia Okanagan Campus, Kelowna, Canada

## Abstract

**Background:**

During the 1900s when industrialization was on the rise in western countries, inflammatory bowel disease (IBD) began to present itself and continually increase over the decades. Now, newly industrialized countries such as India are following this same pattern, and with a population reaching over one billion, India is projected to have one of the highest IBD prevalence worldwide. Furthermore, pediatric diagnoses of IBD are more frequently reported in India and in Indian children living in Canada, suggesting this disease may present differently in those of Indian descent. While the etiology of IBD remains unclear, a gut microbiome that is no longer symbiotic with its host is a key player. However, Indians are one of the least represented in microbiome research, therefore we cannot accurately assess the role of their gut microbiome in IBD. To effectively understand the nature of IBD in Indians, we must first define their gut microbiome.

**Purpose:**

Our study characterizes the microbiome of Indians living in India to explore how it differs from Canadians of European descent.

**Method:**

Stool samples from healthy volunteers (ages 18-55) were collected from Indians in India and Euro-Canadians in Kelowna, BC. Microbial DNA was extracted for 16S sequencing on the Illumina MiSeq platform and QIIME2 was used for microbiome analysis.

**Result(s):**

We will discuss the similarities and differences comparing the gut microbiome of Indians to Euro-Canadians, further highlighting the need for more microbiome research of this demographic.

**Conclusion(s):**

Our research aims to increase representation of Indians in microbiome research in the hopes of improving our knowledge of the predispositions to IBD, which will aid in the information required to develop effective preventive measures. With elevated risk for IBD in Indians residing in Canada, future studies should also aim to analyze if the gut microbiome in Indians change as they migrate and adopt the westernized lifestyle.

**Disclosure of Interest:**

None Declared

